# Expanded Polystyrene Beads Coated with Intumescent Flame Retardant Material to Achieve Fire Safety Standards

**DOI:** 10.3390/polym13162662

**Published:** 2021-08-10

**Authors:** Sangram P. Bhoite, Jonghyuck Kim, Wan Jo, Pravin H. Bhoite, Sawanta S. Mali, Kyu-Hwan Park, Chang-Kook Hong

**Affiliations:** 1 School of Chemical Engineering, Chonnam National University, Gwangju 61186, Korea; sangrambhoite07@gmail.com (S.P.B.); sawantasolar@gmail.com (S.S.M.); 2 HDC HYUNDAI EP R & D Center, Gyeonggi-do 16889, Korea; kimjh1740@hdc-hyundaiep.com (J.K.); jowan@hdc-hyundaiep.com (W.J.); 3 Department of Chemistry, Kisan Veer Mahavidyalaya, Wai 412803, India; bhoiteph@gmail.com

**Keywords:** expanded polystyrene foam, coating, water-based intumescent flame retardant materials, total heat release

## Abstract

The compatibility and coating ratio between flame retardant materials and expanded polystyrene (EPS) foam is a major impediment to achieving satisfactory flame retardant performance. In this study, we prepared a water-based intumescent flame retardant system and methylene diphenyl diisocyanate (MDI)-coated expandable polystyrene microspheres by a simple coating approach. We investigated the compatibility, coating ratio, and fire performance of EPS- and MDI-coated EPS foam using a water-based intumescent flame retardant system. The microscopic study revealed that the water-based intumescent flame retardant materials were successfully incorporated with and without MDI-coated EPS microspheres. The cone calorimeter tests (CCTs) of the MDI-coated EPS containing water-based intumescent flame retardant materials exhibited better flame retardant performance with a lower total heat release (THR) 7.3 MJ/m^2^, peak heat release rate (PHRR) 57.6 kW/m^2^, fire growth rate (FIGRA) 2027.067 W/m^2.^s, and total smoke production (TSP) 0.133 m^2^. Our results demonstrated that the MDI-coated EPS containing water-based intumescent flame retardant materials achieved flame retarding properties as per fire safety standards.

## 1. Introduction

Rapid urbanization has negatively affected the existing thermal balance, resulting in the Urban Heat Island (UHI) effect. Additionally, there has been an increase in energy demands, with the thermal comfort energy consumption for buildings accounting for 30% of the total global energy [1,2]. Therefore, energy savings and emission reduction strategies are fundamental in achieving sustainable urban development [3]. Among the foremost and recently popular strategies for energy saving and thermal balance is enhanced building thermal insulation; consequently, there is increased attention for building insulation materials [4]. 

Currently, commercially available building thermal insulation materials include expanded polystyrene (EPS), rigid polyurethane foam, glass, and rock wool attracted as promising insulating materials [5]. Among them, EPS has attracted more attention as an efficient insulating material in the construction industry, owing to its appealing properties; lightweight, efficient heat insulation, good chemical resistance, and low cost [6]. However, EPS is highly flammable and difficult to make flame retardant because of its interior beehive network, high surface area, and their chemical compositions. Due to its poor flame retardancy, fire safety is still a challenge with EPS as building materials. 

Therefore, to ensure safety, researchers have been exploring methods of enhancing the flame retardation of EPS foam [7,8,9]. Earlier, EPS flame retardation was achieved by coating with halogen flame retardant materials. For example, BASF-manufactured EPS containing halogen flame retardant material foam [10]. However, during the combustion process, halogen flame retardant materials produce toxic substances that are harmful for the environment and people’s health [11,12,13]. This creates an urgent need for halogen-free flame retardant materials [14,15].

Recently, researchers suggested that designing a multiple flame retardant system is a promising strategy to achieve adequate fire performance of the EPS foam. Thus far, intumescent flame retardant (IFR) has emerged as a promising choice as a halogen-free flame retardant for enhancing the performance of flame-retarded EPS due to its superior features, including less smoke and toxicity and high flame retardant efficiency [16,17,18]. The IFRs are categorized into organic IFR (O-IFR) and inorganic IFR (I-IFR). The O-IFR consists of three main fire retardant additives based on their sources, such as an acid source (ammonium polyphosphate, APP), a carbon source (such as pentaerythritol, PER), and a blowing agent (such as melamine, MEL) [19]. 

When exposed to heat, an O-IFR intumescent coating swells up and forms a char layer, between the gas and condensed phases. These compact char layers act as a physical barrier that reduces heat transfer to the substrate layer [20]. As per I-IFRs materials, an expandable graphite (EG) is commonly employed as a carbon char-forming agent, blowing agent as well as a smoke suppressor. During the combustion process, it produces a fluffy intumescent layer that functions as a barrier to heat and gas [21,22,23]. 

In order to enhance flame retardancy, researchers designed an organic-inorganic hybrid flame retardant system (H-IFR). Although, the H-IFR achieves a good flame retardant effect, massive loading is still required to achieve satisfactory flame retardant performance, which hampers its mechanical properties [24,25,26,27]. Many studies confirmed that the combination of an intumescent flame retardant with mineral flame retardants exhibited excellent synergist flame retardant effect [24,25].

Among the available alternatives, mineral flame retardants, such as aluminum hydroxide (Al(OH)_3_ herein ATH) and magnesium hydroxide (Mg(OH)_2_ herein MH), are widely used due to its unique properties, such as low toxicity, minimum corrosion, and low cost [26,27,28,29]. To achieve a satisfactory flame retardant performance, mineral fillers, such as ATH loadings, are relatively high (often between 40% and 70%). As a result, these high loadings hamper its physical and mechanical properties [30,31]. Consequently, the primary objective of flame retardant formulation is to reduce the filler loading while boosting the fire performance [32,33]. 

Therefore, to overcome these challenges, it is important to find an effective and eco-friendly mineral flame retardant materials for EPS. Currently gypsum has attracted attention as an effective and eco-friendly material; it is also cost effective and has high thermal stability. Gypsum is a hydrated mineral, which has fire retardant filler functions similar to ATH and MH. They serve as a heat sink and dilute the combustion gases with the help of releasing water molecules [34,35,36]. However, a single addition of hydrated mineral as a flame retardant is not sufficient to satisfy the flame retardant requirement [37]. 

It is well known that the combination of different additional minerals can enhance flame retardant performance. Currently, calcium carbonate (CaCO_3_) and talc have been extensively been used as flame retarding materials due to their thermal stability, low cost, and availability. However, in a recent study, it was observed that the influence of talc and CaCO_3_ on the fire retardant properties of intumescent composition had a positive effect. During the combustion process, talc and CaCO_3_ can react with phosphate species, which leads to the formation of thermally stable char resulting in increased flame retardant performance [38,39,40].

Generally, these intumescent coating materials are bonded together with the help of polymer binders [41,42]. However, due to environmental concerns, the industrial process is mostly focused on the development of waterborne formulations [43]. Currently, an ethylene vinyl acetate (EVA) binder is commonly used for the preparation of water-based formulation. Despite that, the compatibility between EPS foam and water-based intumescent formulation are key issues due to the viscosity and hydrophobic surface of EPS beads. To overcome these issues, EPS surface modification is a promising solution to enhance its compatibility with water-based intumescent formulation. Currently, 4, 4–methylene diphenyl diisocyanate (MDI) is often used as a binder due to its suitable flashing point (200 °C). MDI can withstand high temperatures and is considered to have a minimal flammability risk [44,45,46,47].

In this work, the MDI was used as binder for EPS bead coating, and EVA as a polymeric binder was used to develop a waterborne intumescent flame retardant formulation. Further, its compatibility and flame resistance performance on EPS and MDI-coated EPS was investigated. The adopted process containing MDI-coated EPS and a waterborne intumescent flame retardant formulation lead to a synergistic effect on the intumescent flame retardant performance. 

The thermal behavior and flame retardancy of the EPS samples were investigated by thermogravimetric analysis (TGA) and cone calorimeter tests (CCT) measurements. In addition, to understand the combustion behavior and flame retardant mechanism of the residues, the char was analyzed by scanning electron microscopy (SEM), Raman, X-ray diffraction (XRD), and X-ray photoelectron spectroscopy (XPS) techniques. To the best of our knowledge, this is the first study to report MDI-coated EPS containing waterborne intumescent flame retardant foam with enhanced flame retardant performance.

## 2. Materials and Method

### 2.1. Materials

The expanded polystyrene (EPS) beads, ammonium polyphosphate (APP, purity > 98%), pentaerythritol (PER, purity 98%), calcium carbonate (CaCO_3_, purity > 98.5%), melamine cyanurate (MC, purity 99%), talc (Whiteness: 94.0 ± 1%, particle size: 11.0 ± 2 μm), and EVA emulsion (G3, solid content 56.5%) were received from HDC HYUNDAI EP Co., Seoul, Korea. Methylene diphenyl diisocyanate (MDI, purity 98%) was purchased from Sigma Aldrich, expandable graphite (EG, purity: 99%, size 270 μm) was purchased from Yuil Chemi Tech Co. Ltd., gypsum (purity >96%) was provided by Namhae chemical corporation, South Korea.

### 2.2. Preparation of the Water-Based Intumescent Flame Retardant Formulation

Table 1 shows the appropriate amount of flame retardant material that was added in distilled water. Then, the solution was homogeneously stirred with a magnetic needle at room temperature for 48 h. The prepared solutions were designated as IFR1, IFR2, and IFR3.

### 2.3. Preparation of MDI-Coated EPS Beads

The MDI-coated EPS beads were prepared by adding and proper mixing with 20 wt.% MDI solution. Then, MDI-coated EPS beads were dried in an oven at 60 °C for 12 h to obtain MDI-coated EPS beads.

### 2.4. Preparation of Flame Retardant MDI-Coated EPS Foam

In a typical experiment, 13 g of EPS were mixed with 39 g of IFR, while, for MDI-coated EPS, 14.5 g of MDI/EPS were used. Note: In this experiment, we found 13 g EPS + 2.6 g MDI resulted in 14.5 g MDI/EPS after complete drying. The mixing ratio of the flame-retardant solution to the EPS beads was optimized from 1:1.5 and 1:3 with the help of char formation, Figure S1. Then, uniformly coated EPS spheres were transferred into a cubic mold and heated at 90 °C in a hot press for 6 h. Cubic shaped EPS foam was carefully removed from the mold and dried in an oven at 60 °C for the next 24 h.

### 2.5. Characterizations

The X-ray diffraction (XRD) measurements were carried out using a D/MAX Uitima IIIXRD spectrometer (PAN analysis, Japan) with Cu Kα line of 1.5410 Å. Scanning electron microscopy: The surface morphological images were recorded by a scanning electron microscope (SEM; S-4700, Hitachi). The elemental information was analyzed using an X-ray photoelectron spectrometer (XPS) (VG Multilab 2000-Thermo Scientific, New York, NU, USA, K-Alpha) with a multi-channel detector that could endure high photonic energies from 0.1 to 3 keV. 

Thermal gravity (TG) analyses of the EPS, MDI/EPS, and IFR/EPS were performed with a heating rate of 10 °C/min from RT up to 800 °C under a nitrogen atmosphere using TA Instruments SDTA 851E. The micro-Raman spectra of the char residue was recorded in the spectral range of 100–3000 cm^−1^ using a micro-Raman spectrometer (inVia Reflex UV Raman microscope (Renishaw, U.K.), KBSI, Gwangju- center) that employed a He-Ne, laser source with an excitation wavelength 633 nm and resolution of 1 cm^−1^ at 15 mW laser power. The Fourier-transform infrared (FTIR) of EPS, MDI, and EPS/MDI (KBr method) was recorded using a Perkin Elmer Spotlight 400 instrument.

#### 2.5.1. Combustion Behavior

A butane spray gun jet was used for the combustion test and applied 5 cm away from the cube-shaped EPS foam for 5 min. Further, the combustion test was monitored by recording digital photographs at each stage.

#### 2.5.2. Cone Calorimetry Test (CCT)

An FTT standard cone calorimeter (Fire Testing Technology Limited, UK) was used to evaluate the flame retardance of the prepared EPS foam samples according to ISO5660 standards under an external heat flux of 50 kW/m^2^ with a specimen dimension of 100 × 100 × 50 mm^3^.

## 3. Results and Discussion

### 3.1. Chemical STRucture

**Figure 1:**
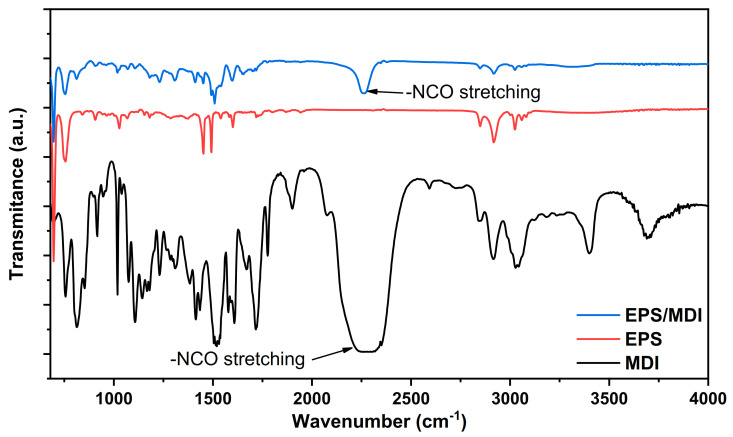
FT-IR spectra of MDI, EPS, and EPS/MDI samples.

The FT-IR spectra of methylene diphenyl diisocyanate (MDI), expanded polystyrene, and MDI-coated EPS shown in Figure 1. In case of the MDI spectrum, the typical strong peaks at 2274, 1770, and 1525 cm^−1^ were assigned to the –NCO group of pure MDI [48]. The neat EPS sample exhibited strong peaks between 3059 cm^−1^ ascribed to C–H stretching vibration, Figure S1. The peak at 2918 cm^−1^ corresponds to the asymmetrical stretching vibration of the CH_2_ group, whereas the peak at 2849 cm^−1^ corresponds to the symmetrical stretching vibration of the CH_2_ group. 

The peaks appearing between 1600–1455 cm^−1^ are associated with the C=C stretching vibration of the benzene ring, whereas the peaks at 1068, 1028, and 757 cm^−1^ were attributed to in-plane and out-of-plane deformation vibration of the benzene ring [49]. However, for the MDI-coated EPS, we observed an additional peak at 2274 cm^−1^ from the stretching vibration of the -NCO group, which indicates the presence of MDI. It is evident that no chemical reaction occurred or new chemical bond was generated during the preparation of this coating material. Therefore, the prepared MDI-coated EPS material had only physical interactions between EPS and MDI.

### 3.2. Microstructural Study

**Figure 2:**
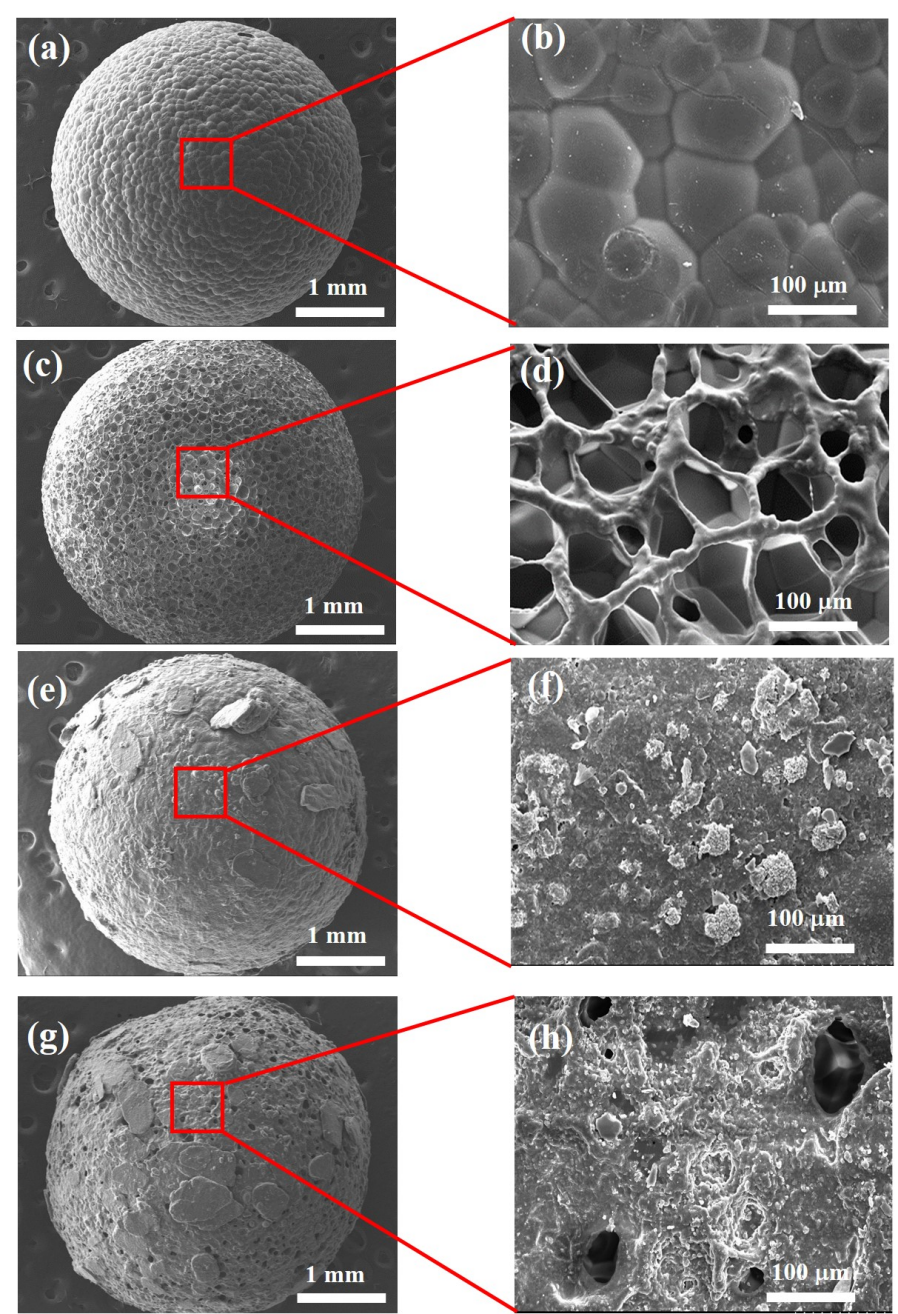
SEM images of the (**a**,**b**) neat EPS, (**c**,**d**) MDI-coated EPS, (**e**,**f**) EPS/IFR3, and (**g**,**h**) EPS/MDI/IFR3 at different magnifications.

The morphological analysis was carried out in order to confirm the quality of uniform coating of MDI on EPS surface and intumescent flame retardant materials. The SEM images of the neat EPS, MDI-coated EPS, EPS/IFR3, and EPS/MDI/IFR3 are shown in Figure 2. The neat EPS had a spherical morphology with a smooth surface, which caused weak compatibility between EPS and flame-retardant materials, Figure 2a,b. Figure 2c,d show that the MDI was uniformly coated on the EPS surface. The smooth surface of the EPS spheres was coated with a mesh network of the MDI coating. However, the smooth surface of the neat EPS was unchanged after the MDI coating, which indicates there was no any chemical reaction on the EPS surface. We believe this this mesh network would enhance the compatibility between EPS and flame retardant materials.

As shown in Figure 2e,f, the EPS sphere was coated with IRF3 material. During the combustion process, intumescent flame retardant materials act as a barrier layer. However, the absence of an MDI layer affected the coating efficiency. As shown in Figure 2g,h, the intumescent retardant materials were uniformly coated on MDI containing EPS spheres. During the combustion process, intumescent flame retardant materials generated compact char, and the shape of the sample remained the same.

### 3.3. Mass Calculation

Mass calculation is an effective quantitative analysis used to evaluate the flame retardant materials coating on the EPS sphere surface. Table 2 displays the mass calculation after coating for EPS/IFR3 and EPS/MDI/IFR3. A simple mass calculation method was used for flame retardant material-coated compounds. We observed that the weight of EPS/IFR3, and EPS/MDI/IFR3 increased from the initial EPS weight of 13 and 14.5 g to 35.5 and 41.90 g, respectively, after coating with flame-retardant materials. 

We found 13 g EPS + 2.6 g MDI resulted in to 14.5 g MDI/EPS after complete drying. To estimate the amount of flame retardant materials used, the weight difference between EPS microspheres before coating and after coating with flame retardant materials was calculated. As shown in Table 2, the EPS/IFR3 sample contained 22.5 gm of flame retardant materials, while the EPS/MDI/IFR3 sample contained 27.4 gm of flame retardant materials. The EPS sphere with a uniform layer of MDI increased the compatibility of the flame retardant materials. 

On the other hand, the EPS/MDI/IFR3 sample contained a higher amount of intumescent flame retardant materials compared with EPS/IFR3. This 27.4 g higher amount of the actual IFR3 loading on the resultant EPS foam arose from the surface modification of the MDI coating. In contrast, our non-modified EPS foam exhibited a lower amount, which clearly indicates a lower amount of loading on the EPS surface. Therefore, MDI-coated EPS with intumescent free flame retardant materials was expected to exhibit better flame retardant performance than EPS coated with flame retardant material.

The apparent densities of EPS, EPS/IFR3, and EPS/MDI/IFR3 were measured and are presented in Table 2. We observed an improved density of the EPS foam after MDI coating. Neat EPS foam exhibited a density of 26 kg/m^3^, which increased up to 71 kg/m^3^ for EPS/IFR3 and 83 kg/m^3^ for EPS/MDI/IFR3. This increased density owing to the MDI binder enhanced the compatibility between EPS and the water-based flame retardant formulation. This improved density may lead to increased mechanical properties of the EPS foam. However, further investigation on the mechanical properties of these developed EPS is needed.

### 3.4. Thermogravimetric Analysis (TGA)

**Figure 3:**
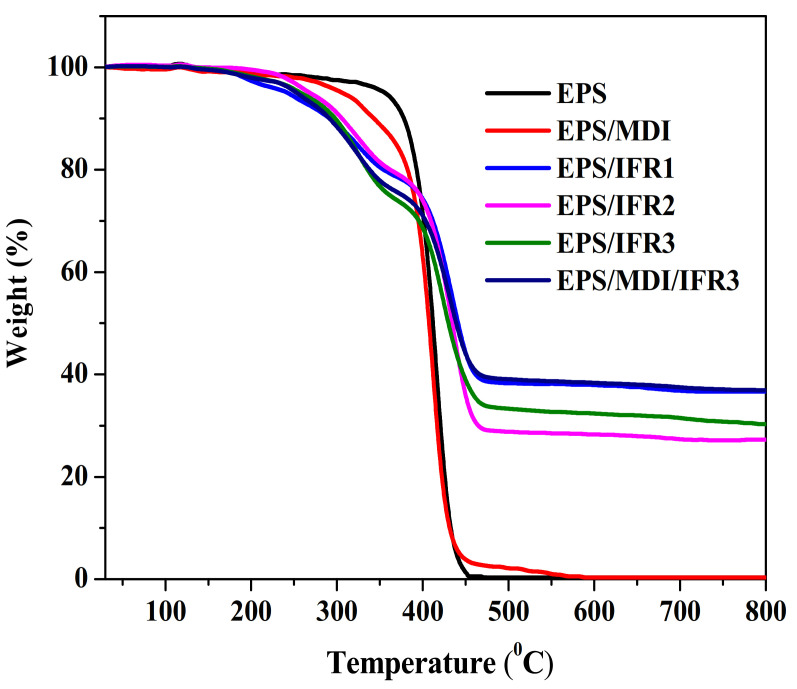
TGA curves of the EPS and flame retardant EPS samples under a nitrogen atmosphere at a heating rate of 10 °C/min.

TGA is reliably used to measure the thermal degradation behaviors of the flame retardant materials. Considering the fire safety standards, the thermal resistance of flame retarding materials is important and plays a crucial role in the active application and viability of flame retarding materials. Figure 3 shows the TGA curves of the EPS and flame retardant EPS. The TGA plot display the EPS showing the initial weight loss temperature at 340 °C and also exhibiting rapid decomposition steps at 450 °C with no residue left after decomposition. However, after the coating of MDI, the thermal stability decreased compared to neat EPS, and no residue was left after decomposition. 

The final char residue weight of flame retardant EPS varied with different flame retardant combinations, indicating that APP significantly accelerated the decomposition of different flame retardant combinations. The initial weight loss of flame retardant EPS showed a similar decomposition behavior, due to decomposition of the gypsum starting from 100 to 150 °C corresponding to the evaporation of water molecules [34,36,50].

For the EPS/IFR1 sample, the major weight loss was observed between 180–450 °C. At the temperature of 180 °C, the EG begin to decompose and release sulfur dioxide [51]. Meanwhile, APP began decomposing to form polyphosphoric acid. Further, the decomposition step was attributed to a possible esterification reaction between acid and PER. With increasing temperature, the degradation of the ester began to form char [52]. Moreover, the talc and calcium carbonate could react with a polyphosphate network leading to a reduced degradation rate and improved heat stability.

On the other hand, the TGA curve of EPS/IFR2 sample shows that EG began decomposing at 180 °C. The major weight loss was observed between 210 to 450 °C due to the interactions between APP and MC. The APP started decomposing and releasing NH_3_ and water to form polyphosphoric acid. Enhancing the flame retardant properties, MC began to decompose between 290 to 420 °C, and released incombustible nitrogen gas that could further expand and inflate the formed char [53]. 

The further decomposition stage was attributed due to the decomposition of talc and calcium carbonate. The TGA plot displays the char residue of EPS/IFR2 sample decreased compare to the EPS/IFR1 sample, which may be due to MC acting as a blowing agent as it has a limited char forming tendency. For the EPS/IFR3 sample, the major weight loss was observed in the 180 to 410 °C region. The EG began decomposing and released inflammable gases. The APP started to decompose, releasing water and ammonia to form polyphosphoric acid. The esterification reaction occurred between acid and PER between 210 to 300 °C. 

Boosting the flame retardant properties, MC began to decompose around 300–400 °C producing incombustible nitrogen gases that swelled and inflated the char. With increasing temperature, the talc and calcium carbonate reacted with the polyphosphate network produce thermally stable char [38,54]. For the EPS/MDI/IFR3 sample, MDI plays the role of a binder, which led to improved compatibility between EPS and the flame retardant materials. The TGA curve displays a similar thermal degradation profile as the EPS/IFR3 sample except for the small weight loss observed in the 270–290 °C region. 

**Scheme 1:**
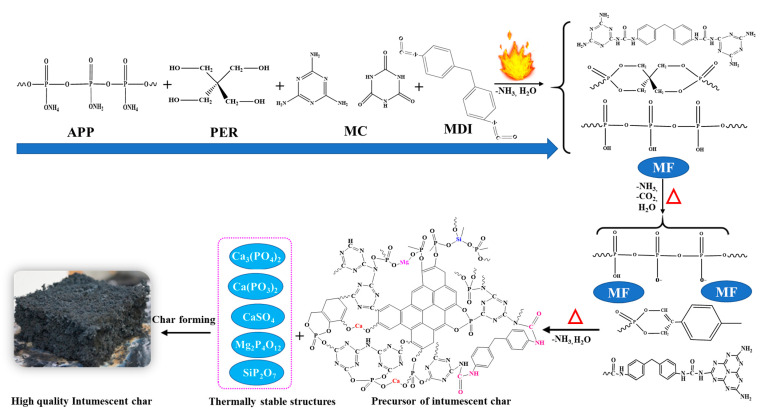
Possible flame retardant mechanism during the combustion process. Here, mineral filler (MF) contains CaCO_3_ and talc.

This might be due to interactions between MC and iscocynate. The -NH_2_ group from MC reacted with the -NCO group of MDI, which can suppress the amount of smoke release. Our CCT results further revealed the interaction between MC and MDI [55]. The TGA curve displays the char residue weight increase compared to the EPS/IFR3 sample. The possible reaction mechanism during the combustion process is shown in Scheme 1.

### 3.5. Combustion Behavior

**Figure 4:**
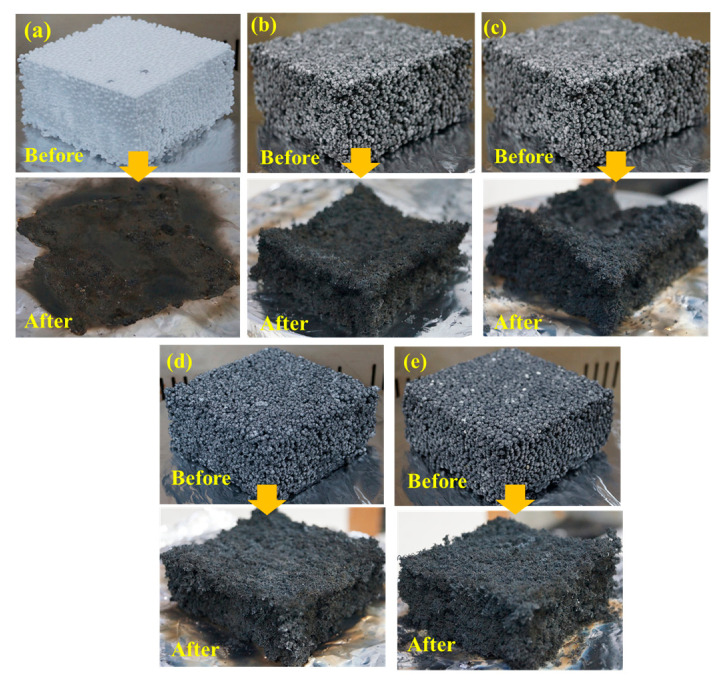
The digital photographs of combustion phenomenon of (**a**) neat EPS, (**b**) EPS/IFR1, (**c**) EPS/IFR2, (**d**) EPS/IFR3, and (**e**) EPS/MDI/IFR3.

The coating ratio between EPS and flame retardant materials play a key role in achieving satisfactory flame retardancy. Incorporating the higher amount of flame retardant material into EPS conciliate the quality of the foam. Therefore, optimization of amount of the flame retardant materials is essential. To optimize the amount of the flame retardant materials, we prepared different combinations of water-based intumescent flame retardant materials with EPS. Digital photographs of the samples during the combustion test are shown in Figure 4.

Figure 4a shows a photograph of the neat EPS sample, which produced a large amount of smoky and sooty flame, and no char was left after combustion. The sample with a coating ratio of 1:1.5 had poor expansion properties resulting in discontinuous and broken char (Figure S2). In addition, the sample with a coating ratio of 1:3 generated enough char after the combustion process. In Figure 4b,c, the EPS/IFR1 and EPS/IFR2 samples produced more char compared to neat EPS but failed to generate continuous and compact char during the combustion process. In contrast, Figure 4d,e shows that the EPS/IFR3 and EPS/MDI/IFR3 samples generated a well-expanded and compact char layer after combustion. This compact char layer acted as a protecting shield, resulting in an enhanced flame retardant performance of the EPS foam.

### 3.6. Cone Calorimetry Analysis

**Figure 5:**
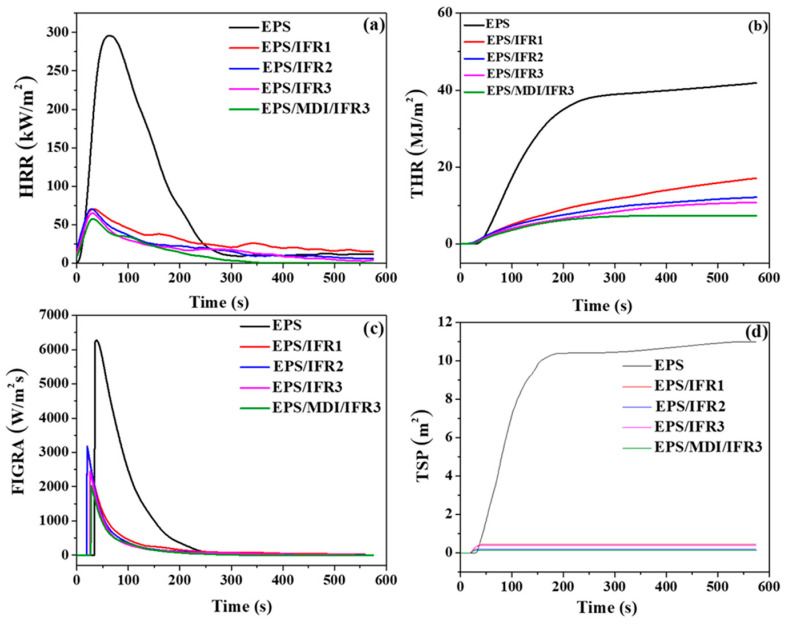
Cone calorimeter curves of neat EPS, EPS/IFR1, EPS/IFR2, EPS/IFR3, and EPS/MDI/IFR3 samples: (**a**) PHRR, (**b**) THR, (**c**) FIGRA, and (**d**) TSP.

The cone calorimeter test is a scientific and effective method used to investigate the combustion behavior and flame retardant mechanism of EPS foam for onsite applications [56]. Figure 5 illustrates the peak heat released rate (PHRR), total heat release (THR), fire growth rate (FIGRA) and total smoke production (TSP) of neat EPS, EPS/IFR1 EPS/IFR2 EPS/IFR3, and EPS/MDI/IFR3. Detailed parameters are provided in Table 3. The PHRR and THR curves of neat EPS are shown in Figure 5a,b. The result indicates that the neat EPS quickly burned after ignition with a high PHRR of 295.5 kW/m^2^ and THR of 42.3 MJ/m^2^ values. 

However, after incorporating a water-based intumescent flame retardant into the EPS foam, the EPS/IFR1 and EPS/IFR2 samples showed an obvious reduction in the PHRR and THR values as compared to neat EPS, from 70.2 and 70.3 kW/m^2^ to 17.6 and 12.3 MJ/m^2,^ respectively. This is a good indication that the water-based intumescent flame retardant reduced the PHRR and THR values for the EPS/IFR1 and EPS/IFR2 samples. We observed that the PHRR and THR values were drastically reduced to 65 kW/m^2^ and 10.9 MJ/m^2^ for EPS/IFR3. The reduced values indicate the synergistic effect of an optimized combination of flame retardant materials, which produced a large amount of compact char. This demonstrated that the EPS/IFR3 combination sample acted as a more effective barrier layer as compared to the EPS/IFR1 and EPS/IFR2 samples.

Due to less compatibility between EPS and water-based intumescent flame retardant materials, the EPS/IFR3 sample did not satisfy the fire safety norm. The EPS/MDI/IFR3 sample produced the lowest PHRR and THR values of 57.6 kW/m^2^ and 7.3 MJ/m^2^, respectively, when compared to the other samples. This illustrates that MDI could enhance the compatibility between EPS and flame retardant materials, thus, leading to lower PHRR and THR values [57]. 

These values are optimal based on fire safety standards. Figure 5c shows the FIGRA results evaluation regarding the burning properties of the materials. The FIGRA value of the neat EPS, EPS/IFR1, EPS/IFR2, EPS/IFR3, and EPS/MDI/IFR3 samples were 6274.0, 3086.2, 3176.7, 2441.9, and 2027.0 W/m^2^s, respectively. A lower FIGRA value indicates effective fire safety, and this means that there would be enough time to evacuate people or to extinguish the fire [58].

The generation of smoke in a fire is one of the main causes of human death, either directly by suffocation or by the inhalation of poisonous gases. The TSP curves demonstrated that, after the flame retardant coating, the TSP value greatly decreased from 10.99 m^2^ for neat EPS to 0.457, 0.196, 0.394, and 0.133 m^2^ for EPS/IFR1, EPS/IFR2, EPS/IFR3, and EPS/MDI/IFR3, respectively as shown in Figure 5d and Table 3. The substantial reduction in TSP demonstrated that the water-based intumescent flame retardant coatings effectively suppressed smoke formation, which would remarkably increase the chances of people escaping in a fire [59].

### 3.7. Char Residue Analysis

**Figure 6:**
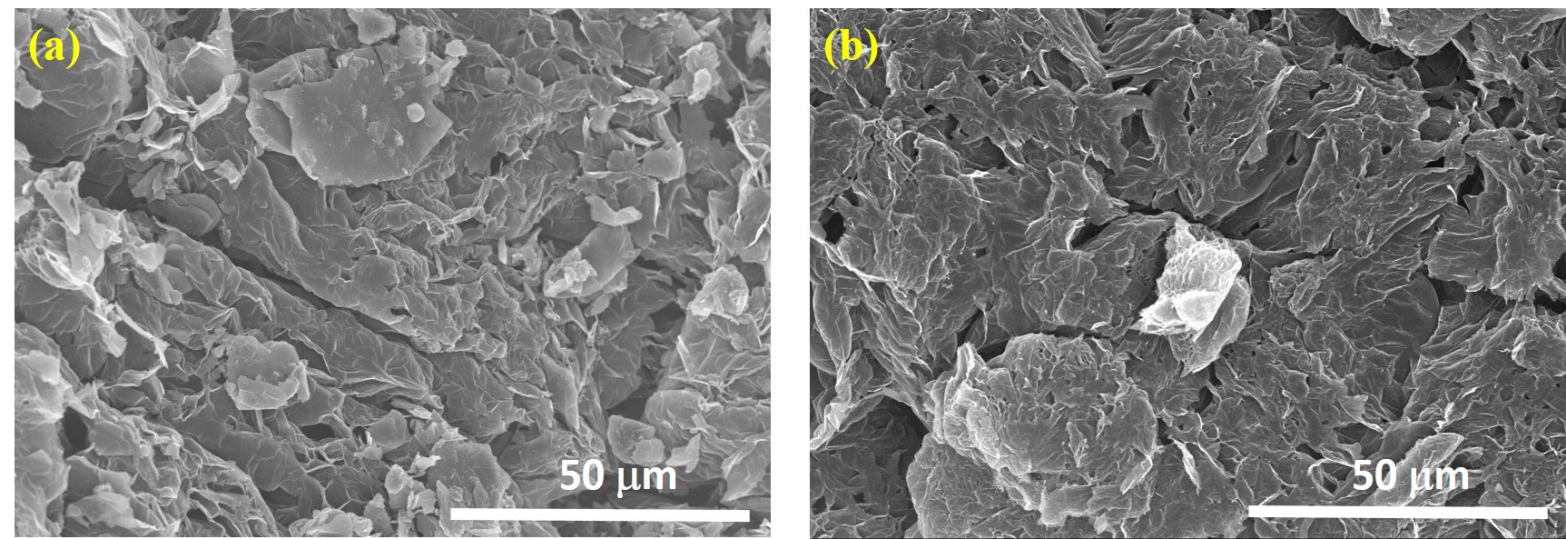
SEM images of the char residue after the CCT test (**a**) EPS/IFR3 and (**b**) EPS/MDI/IFR3.

Figure 6 presents SEM micrographs of the char residue that were obtained after the CCT test. Figure 6a shows the SEM micrograph of the residual char corresponding to the EPS/IFR3 sample, revealing an incompact and discontinuous surface formed after the combustion process. In contrast, the SEM micrograph of the residual char of the EPS/MDI/IFR3 sample exhibits compact and continuous char formation compared to the EPS/IFR3 sample. This compact char acts as effective, protective barrier layers during combustion. Therefore, EPS/MDI/IFR3 showed excellent fire performance compared with the EPS/IFR3 sample.

**Figure 7:**
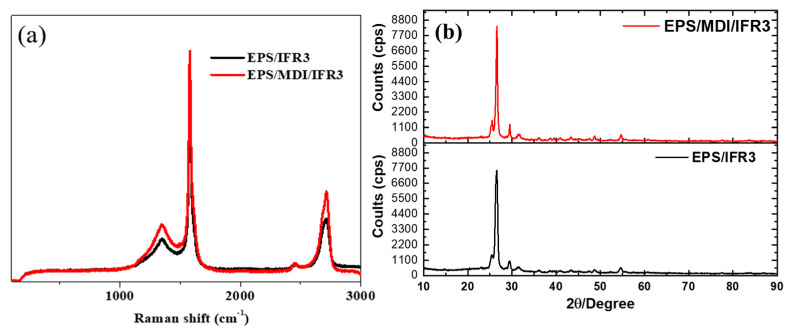
Char residue analysis. (**a**) Raman spectra and (**b**) XRD patterns of the char residue of EPS/IFR3 and EPS/MDI/IFR3.

The Raman spectra was obtained to characterize the graphitization degree of carbonaceous char material [60,61]. As portrayed in Figure 7a, the Raman spectra of char shows a carbon signal with the D band at ca. 1360 cm^−1^ and G band at ca.1580 cm^−1^. As a result, the D and G bands have different intensities, which are associated with the amorphous and graphitized carbon content [62]. The effectiveness of the flame-retardancy depends not only on the amount of char residues present but also on the quality of those residues.

A lower I_D_/I_G_ ratio indicates a more stable char structure and more extensive graphitization results in higher flame retarding properties. The calculated I_D_/I_G_ ratio for EPS/MDI/IFR3 (0.50) was less than that of EPS/IFR3 (0.54), indicating an extensive graphitization degree residue. The MDI-coated EPS could improve the compatibility with IFR, thus, leading to facilitating the char graphitization after combustion. In addition, the XRD patterns of the EPS/IFR3 and EPS/MDI/IFR3 residue char of samples was carried out to identify the crystallinity, Figure 7b. The XRD pattern of the EPS/MDI/IFR3 sample showed higher peak intensity due to improved crystallinity, which arose due to better compatibility between EPS and the flame retardant materials, which is consistent with our Raman analysis.

#### Chemical Composition Char Residue

**Figure 8:**
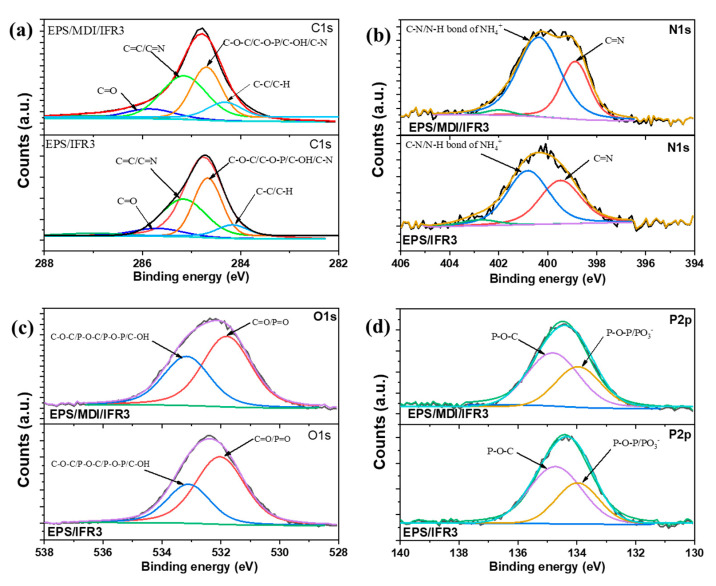
High resolution XPS spectra of (**a**) C1s, (**b**) N1s, (**c**) O1s, and (**d**) P2p core-level spectra of char residues for the EPS/IFR3 and EPS/MDI/IFR3 samples after the cone calorimeter test.

After the cone calorimeter test, the chemical compositions of the char residues were further analyzed using XPS. The detailed composition is shown in Table 4 and Figure 8. The XPS spectra of the char residues for EPS/IFR3 and EPS/MDI/IFR3 after the CCT test is shown in Figure S3. EPS/MDI/IFR3 had greater P/C and N/C ratios than EPS/IFR3, which indicates that more P- and N-containing compounds were formed in the char residue. According to Yuan et al., more P and N remaining in the char layer is beneficial to produce a more compact char layer that efficiently limits heat and mass transfer [63]. 

Furthermore, the C/O ratio of the EPS/MDI/IFR3 was larger than that of EPS/IFR3, indicating that the anti-oxidation property of char was enhanced. EPS/MDI/IFR3 had a larger carbon content in the residue char compared with EPS/IFR3, which may be due to the high content of flame retardant materials. Figure 8a shows C1s core-level spectra exhibiting the binding energy peaks at around 284.5, 285.0, and 286.3 eV assigned to C–C, C–H, and C–O, C–N, or C=N, C=O groups, respectively [64]. 

EPS/MDI/IF3 had significantly higher C–O and C–N contents than did EPS/IFR3, which indicates that more cross-linking frameworks including C–O or C–N predominated in the char of EPS/MDI/IF3. This could be due to the high content of intumescent formulation as stated in Table 4. As shown in Figure 8b, the N1s core–level spectra exhibited C=N, C–N, or N–H bonding at 400.83 eV and 399.46 eV [65]. If we compare the N1S peak intensity, the EPS/MDI/IFR3 sample exhibited higher intensity over EPS/IFR3. 

This is evidence of a higher cross-linked framework of C–N formed in EPS/MDI/IFR3. Figure 8c corresponds to the O1s core-level spectra, with the binding energy peak at 532 to 533.15 eV allocated to C=O or P=O and C–O–C/P–O–P/C–OH or P–O–C groups. As stated in Table 4, we observed that the oxygen content was decreased in the EPS/MDI/IFR3 sample. The P2p core-level spectra in Figure 8d shows the binding energy peak at 133.94 eV associated with P–O–P or PO_3_ groups. 

The peak at 134.75 eV is allocated to a P–C–O group [66]. The EPS/MDI/IFR3 showed a high percent of bands at 134.75 eV as compared to EPS/IFR3, which demonstrates that more cross linked frameworks predominated in the char. From the above result, it is clear that the MDI binder enhanced the compatibility between water-based intumescent formulation and EPS foam, which led to more cross-linked structure being formed.

### 3.8. Possible Reaction Mechanism

**Figure 9:**
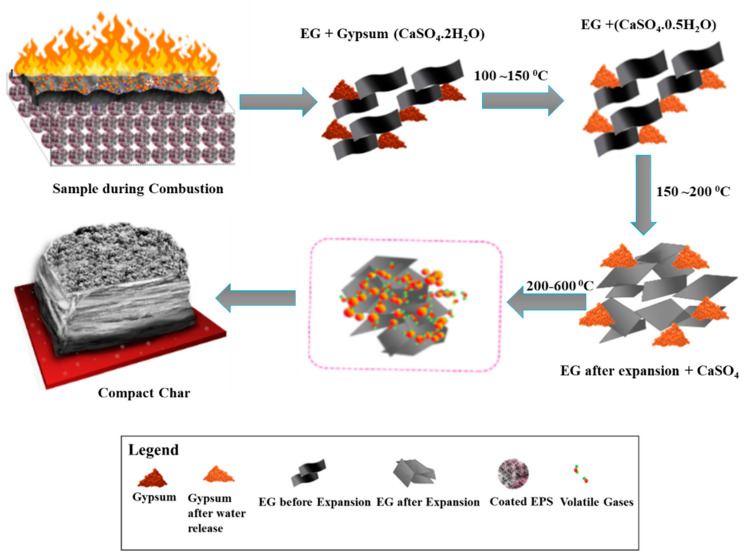
The combustion mechanism of the EPS/MDI/IFR3 sample.

Based on the above analysis, the possible flame retardant mechanism of the EPS/MDI/IFR3 sample is shown in Figure 9. During the combustion process, gypsum (CaSO_4_.2H_2_O) released water molecules and formed anhydrous calcium sulfate (CaSO_4_). At the same time, EG began expanding and releasing nonflammable gases, such as CO_2,_ SO_2_ [67,68]. These nonflammable gases diluted the oxygen and absorbed heat, leading to a decreased heat release rate. With the increasing temperature, APP decomposed to generate incombustible gases (NH_3_ and H_2_O), orthophosphate, and phosphoric acid. The esterification reaction took place between phosphoric acid and PER under milder conditions and reduced the combustion intensity. 

Simultaneously, interactions between MC and MDI generated a polymeric network that produced a positive effect on the barrier. With increasing temperature, this began decomposing and releasing nonflammable gasses (NH_3_, CO_2,_ and H_2_O) that diluted the oxygen concentration around the flame and swelled the precursor of intumescent char. This led to prevention of the spreading flame further and suppressed the smoke release [53,69]. At higher temperatures, the talc and CaCO_3_ reacted with polyphosphoric acid resulting in the formation of a thermally stable silicon phosphate and calcium phosphate containing intumescent char that produced a positive effect on the anti-oxidation properties of the char. As a result, more compact char was formed, which led to effectively boosting the flame resistance performance of the EPS foam.

## 4. Conclusions

In conclusion, we demonstrated the role of MDI as a binder and coating on expanded PS beads to improve the compatibility between EPS and water-based flame retardant formulations. Our SEM and FTIR analysis confirmed that the MDI was fully covered on the expanded PS beads uniformly and that there was no chemical reaction occurring between the EPS beads and MDI binder. The TGA analysis revealed that MDI-coated EPS exhibited more thermal stability compared with the non-coated sample, which indicates a key role of MDI and IFR in its thermal stability behavior. The CCT results exhibited that the EPS/MDI/IFR3 sample provided lower THR (7.3 MJ/m^2^) and PHRR (57.6 kW/m^2^) values as compared to the other flame retardant EPS foams. 

The lower FIGRA and TSP values demonstrated the key role of MC and MDI interaction. The char residue analysis confirmed that the MDI binder enhanced its compatibility between the EPS and water-based flame retardant formulation, which led to more compact char being formed after combustion. Our Raman and XRD analyses of the char confirmed increased graphitization and crystallization, which may provide better thermal stability and reduce the heat release rate. Our findings provide a framework and insight into the development of EPS materials that fulfill fire safety standards.

## Figures and Tables

**Table 1:** The preparative parameters of water-based intumescent flame retarding formulations. Note: In this composition, we fixed 55 g EVA, 9 g Gypsum, 35 g EG, 1g Talc, and 15 g APP in 100 mL of water.

Sample Code	PER (g)	CaCO_3_ (g)	MC (g)
IFR1	7.5	7.5	0
IFR2	0	7.5	7.5
IFR3	5	5	5

**Table 2:** Mass of the flame-retardant materials coated on the EPS surface.

Sample Code	Initial Wt. of EPS (g)	Final Weight(g) after Drying (1:3 ratio)	Wt. of IFR Coated on EPS (g)	Density (kg/m^3^)
EPS	13	-	-	26
EPS/IFR3	13	35.5	22.5	71
EPS/MDI/IFR3	14.5	41.90	27.4	83

**Table 3:** The cone calorimeter test results for different EPS foams.

Sample Code	PHRR (kW/m^2^)	THR (MJ/m^2^)	FIGRA (W/m^−2^s)	TSP (m^2^)
EPS	295.5	42.3	6274.0	10.99
EPS/IRF1	70.2	17.6	3086.2	0.457
EPS/IRF2	70.3	12.3	3176.7	0.196
EPS/IRF3	65	10.9	2441.9	0.394
EPS/MDI/IFR3	57.6	7.3	2027.0	0.133

**Table 4:** X-ray photoelectron spectroscopy data of the char residues for EPS/IFR3 and PS/MDI/IFR3.

Sample Name	C%	O%	N%	P%	Ca%	S%	Mg%	Si%	C/O	P/C	N/C
EPS/IFR3	86.06	8.96	1.31	0.78	0.33	0.15	0.14	2.26	9.60	0.009	0.015
EPS/MDI/IFR3	88.61	7.6	2.7	1.31	0.4	0.24	0.13	0.01	11.39	0.015	0.031

## Data Availability

All data has been provided in within this manuscript.

## Supplementary Materials

The following are available online at https://www.mdpi.com/article/10.3390/polym13162662/s1, Figure S1. Detailed FTIR analysis of MDI, EPS and MDI coated EPS. Note: Colour codes are same for all graphs as per main text. Figure S2. The digital photographs of the combustion phenomenon of (S1) EPS/IFR1 (S2) EPS/IFR2 and (S3) EPS/IFR3 (coating ratio 1:1.5). Figure S3. XPS survey spectra of char residues for EPS/IFR3 and EPS/MDI/IFR3 after cone calorimeter test.
