# Supplementary material for: Expanded Polystyrene Beads Coated with Intumescent Flame Retardant Material to Achieve Fire Safety Standards
Source: Polymers (Basel). 2021 Aug 10;13(16):2662. doi: 10.3390/polym13162662 (PMC8398555; doi:10.3390/polym13162662)
Supplement: Supplementary file 1 [file polymers-13-02662-s001.zip › polymers-1249098-supplementary.pdf]

polymers

MDPI

Article

Expanded Polystyrene Beads Coated with Intumescent Flame Retardant Material to Achieve Fire Safety Standards

Sangram P. Bhoite [1,†] Jonghyuck Kim [2,†], Wan Jo [2], Pravin H. Bhoite [3], Sawanta S. Mali [1], Kyu-Hwan Park [2,*] and Chang Kook Hong [1,*]

1 School of Chemical Engineering, Chonnam National University, Gwangju 61186, Korea; sangrambhoite07@gmail.com (S.P.B.); sawantasolar@gmail.com (S.S.M.)
2 HDC HYUNDAI EP R & D Center, Yongin-si, Gyeonggi-do 463811, Korea; kimjh1740@hdc-hyundaiep.com (J.K.); jowan@hdc-hyundaiep.com (W.J.)
3 Department of Chemistry, Kisan Veer Mahavidyalaya, Wai 412803, MS, India; bhoiteph@gmail.com
* Correspondence: kyu@hdc-hyundaiep.com (K.-H.P.); hongck@jnu.ac.kr (C.K.H.)
† These authors contributed equally to this work.

Citation: Bhoite, S.P.; Kim, J.; Jo, W.; Bhoite, P.H.; Mali, S.S.; Park, K.-H.; Hong, C.K. Expanded Polystyrene Beads Coated with Intumescent Flame Retardant Material to Achieve Fire Safety Standards. *Polymers* **2021**, *1*, 6266. https://doi.org/10.3390/polym1316266

Academic Editor: Vinayak G Parale, Hyung-Ho Park and Varsha D. Phadtare

Received: 21 May 2021
Accepted: 6 August 2021
Published: 10 August 2021

Publisher's Note: MDPI stays neutral with regard to jurisdictional claims in published maps and institutional affiliations.

Copyright: © 2021 by the authors. Licensee MDPI, Basel, Switzerland. This article is an open access article distributed under the terms and conditions of the Creative Commons Attribution (CC BY) license (http://creativecommons.org/licenses/by/4.0/).

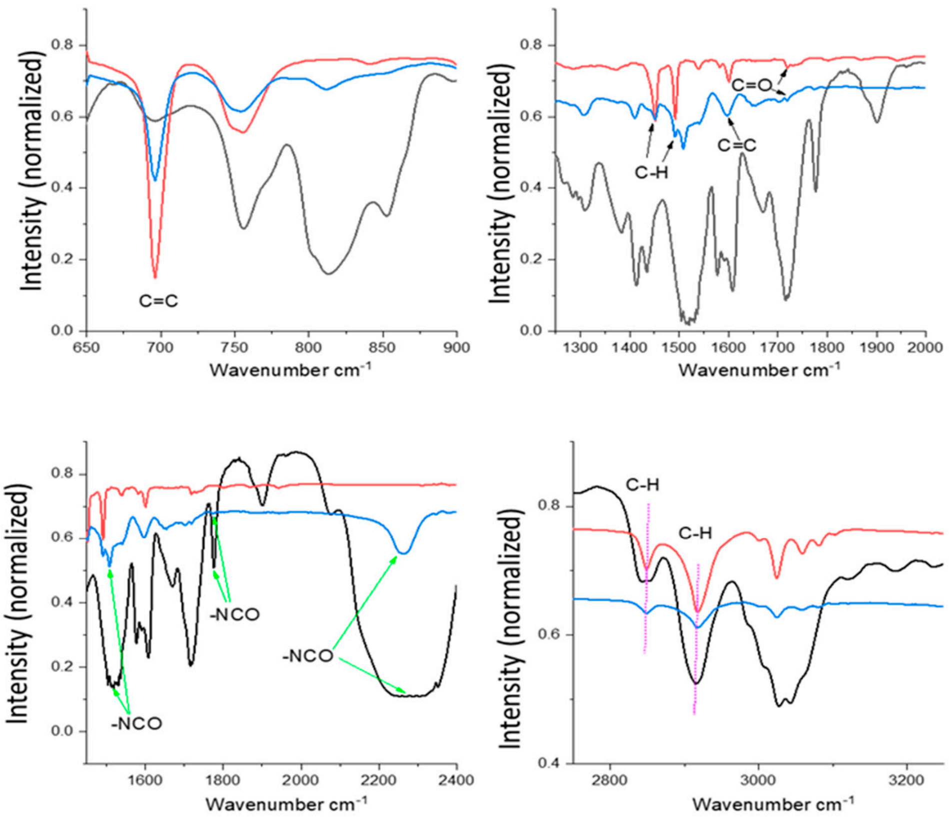

Figure S1. Detailed FTIR analysis of MDI, EPS and MDI coated EPS. Note: Colour codes are same for all graphs as per main text.

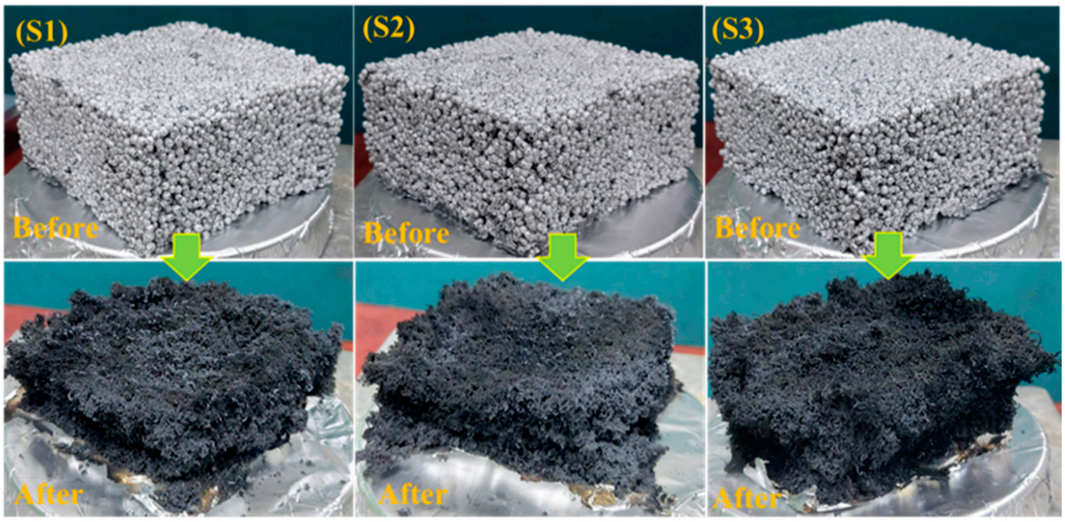

Figure S2. The digital photographs of the combustion phenomenon of (S1) EPS/IFR1 (S2) EPS/IFR2 and (S3) EPS/IFR3 (coating ratio 1:1.5).

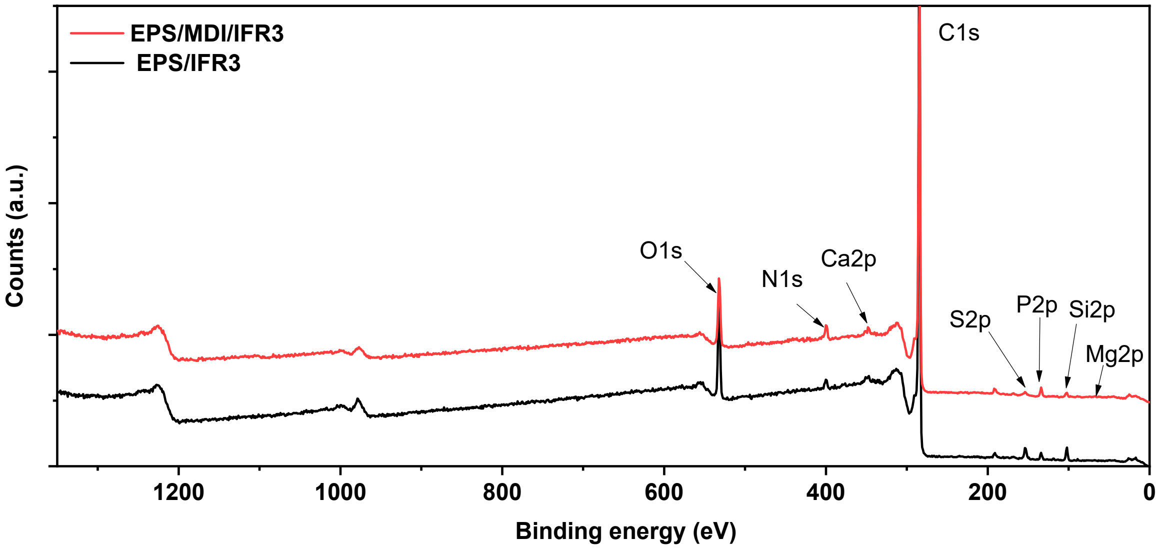

Figure S3. XPS survey spectra of char residues for EPS/IFR3 and EPS/MDI/IFR3 after cone calorimeter test.
